# The Application of Dendritic Cells Vaccines in Tumor Therapy and Their Combination with Biomimetic Nanoparticles

**DOI:** 10.3390/vaccines13040337

**Published:** 2025-03-21

**Authors:** Tong Zhu, Yuexin Li, Yutao Wang, Danyang Li

**Affiliations:** 1 Panjin Central Hospital, Panjin 124010, China; 2019122198@cmu.edu.cn; 2 Department of Pharmacy, Harbin Medical University Cancer Hospital, Harbin 150081, China; liyuexin95@hrbmu.edu.cn; 3 Department of Urology, Peking Union Medical College Hospital, Chinese Academy of Medical Sciences and Peking Union Medical College, Dongcheng, Beijing 100000, China; 4 Department of Pharmacology, College of Pharmacy, Harbin Medical University, Harbin 150081, China

**Keywords:** dendritic cell, DC vaccines, TME, biomimetic nanoparticles

## Abstract

Dendritic cells (DCs) act as a bridge between innate and adaptive immunity by presenting antigens to effector immune cells and have shown broad application potential in tumor immunotherapy. However, the clinical translation of DC vaccines encounters significant challenges, such as the immunosuppressive tumor microenvironment (TME) and the sub-optimal DC function and vaccine efficacy in vivo. In this review, our investigation has uncovered the latest developments in DC vaccines and their potential in cancer immunotherapy, with a special emphasis on the integration of nanotechnology. Several types of nanomaterials, including protein cage nanoparticles (NPs), biomimetic NPs, and targeted multifunctional NPs, have been developed to enhance the antigen presentation ability of DCs and their stimulatory effects on T cells. In addition, we have also summarized the synergistic anti-cancer effects of DC vaccines with immune checkpoint inhibitors, chemotherapy, and radiotherapy. In addition, recent advances in nanotechnology have made it possible to develop novel biomarkers that can enhance the antigen presentation capacity of DCs and stimulate T cells. These biomarkers not only improve the accuracy and precision of DC vaccine design but also provide new insights into understanding the mechanisms of the DC-mediated immune response. Despite challenges pertaining to technical complexities and individual adaptation in the design and production of DC vaccines, personalized immunotherapy based on DCs is expected to become an important part of cancer treatment with rapid developments in biotechnology and immunology. This review provides new perspectives and potential solutions for the optimal design and application of DC vaccines in cancer therapy.

## 1. Introduction

The immune system is pivotal in both the prevention and treatment of cancer. Antigen-presenting cells (APCs), particularly dendritic cells (DCs), stimulate adaptive immune responses by processing antigens and presenting antigenic peptides to effector T cells via MHC molecules. Over the past few years, vaccines based on DCs have become increasingly popular for cancer immunotherapy, owing to their precise targeting and excellent control [1]. However, despite the ability of DC vaccines to elicit specific anti-tumor immune responses, their clinical application is constrained by the immunosuppressive nature of the tumor microenvironment (TME), the sub-optimal intensity of vaccine-induced immune responses, significant differences among individual patients, and technical challenges in production. TME represents a complex and intricate setting that plays a pivotal role in the advancement of cancer and its response to therapy. This environment encompasses a variety of components, such as immune cells, stromal cells, and signaling molecules that have the potential to hinder immune system activity. Immunotherapy approaches, including DC vaccines, are designed to activate the body’s defenses against tumors. The presence of immunosuppressive cytokines, such as transforming growth factor β (TGF-β) and interleukin 10 (IL-10), as well as that of regulatory T cells (Tregs) and myeloid-derived suppressor cells (MDSCs) in the TME, significantly weakens the antigen presentation ability of DCs and the subsequent activation of T cells [2]. In addition, traditional DC vaccines are usually loaded with a single tumor-specific antigen or limited immune adjuvants, which results in sub-optimal immune responses due to tumor heterogeneity. Therefore, the current research directions in DC-based immunotherapy are aimed at optimizing the antigen-loading strategy of DC vaccines, enhancing the diversity of immune adjuvants, and overcoming tumor immune escape through combined therapies.

In recent times, the emergence of nanotechnology has introduced innovative instruments for creating DC vaccines. The utilization of nanomaterials is prevalent in improving antigen delivery and boosting immune responses thanks to their distinctive physicochemical characteristics, including a large specific surface area, compatibility with biological systems, and multifunctional capabilities [3]. For example, protein cage nanoparticles (NPs) can efficiently deliver tumor antigens to the target DCs and improve antigen presentation, while biomimetic NPs can significantly improve the efficacy and specificity of DC vaccines by modifying the cell surface molecules. Furthermore, multifunctional NPs have the capability to concurrently transport antigens, adjuvants, and immunomodulatory agents, thereby providing an opportunity for multi-tiered stimulation of DC vaccines. The integration of DC vaccines alongside other cancer therapies presents a hopeful therapeutic avenue. For example, immune checkpoint inhibitors, including PD-1/PD-L1 antagonists, can work in synergy with DC vaccines by counteracting the immunosuppressive environment created by tumors [4]. Additionally, it has been demonstrated that the effectiveness of DC vaccines can be improved by chemotherapy and radiotherapy as these treatments facilitate the release of tumor antigens and attract immune cells [5].

In this review, we have summarized the latest progress in DC vaccines for cancer immunotherapy, with a particular focus on the application prospects of nanotechnology. We evaluated the safety and efficacy of DCs immunotherapy in patients with refractory solid malignancies. Meanwhile, we demonstrated clinical and immunologic responses in indolent B-cell lymphoma patients vaccinated with autologous tumor-loaded DCs. Finally, we conducted a phase II randomized trial comparing autologous tumor lysate DC therapy with the best supportive care in pre-treated advanced colorectal cancer patients. Our objective is to deliver a scientific foundation and practical insights for improving the design of DC vaccines and formulating combined treatment approaches for cancer.

## 2. The Characteristic of DCs

### 2.1. Definition and Properties of DCs

DCs represent a category of highly specialized immune cells responsible for capturing, processing, and presenting both exogenous and self-antigens to T lymphocytes, which consequently triggers specific immune responses [6]. The characteristic dendritic projections of these cells facilitate efficient antigen capture by increasing their surface area. The DCs essentially act as a “bridge” connecting the innate and adaptive immune systems by detecting foreign antigens and triggering suitable T cell responses. Additionally, DCs also play an important role in maintaining immune tolerance [7].

Based on their function and origin, DCs are primarily categorized as classical DCs (cDCs), plasmacytoid DCs (pDCs), and monocyte-derived DCs (moDCs) [8,9]. The cDCs are primarily located in peripheral tissues like the skin, lungs, and intestines, where they trigger immune responses against infections by detecting pathogen-associated molecular patterns (PAMPs) [10]. Furthermore, cDCs have an important function in the immune response against tumors. These cDCs can be classified into two types, cDC1 and cDC2, depending on their surface markers. The cDC1 subtype is mainly responsible for triggering cell-mediated immune responses, particularly CD8+ T cell responses, by facilitating cross-presentation of antigens. Conversely, cDC2 is primarily involved in enhancing humoral immune responses, like those from CD4+ T cells, and is vital for the regulation of allergic and autoimmune conditions [11]. The pDCs are distributed in the peripheral blood and lymphoid organs and play a vital role during the early stages of viral infection [12,13]. Following recognition of viral RNA or DNA, pDCs activate the toll-like receptors (TLRs) and subsequently secrete large amounts of interferons (IFNs) to mount an anti-viral response [14]. The moDCs differentiate from monocytes in response to inflammatory stimuli or tissue damage and migrate to the affected sites via the bloodstream and lymphatic system, where they present antigens to the T cell [15,16,17].

### 2.2. The Origin and Differentiation of DCs

During the embryonic stage, DCs primarily arise from the blood islands or progenitor cells in the bone marrow [18]. Although not entirely mature, embryonic DCs act as the initial barrier against pathogens and carry out immune monitoring in peripheral tissues [19]. Furthermore, embryonically derived DCs are generally not stimulated by external pathogens but exist in a “mature resting state” that is crucial for mounting a response against potential pathogen invasions and for establishing and maintaining immune tolerance [20].

**Figure 1:**
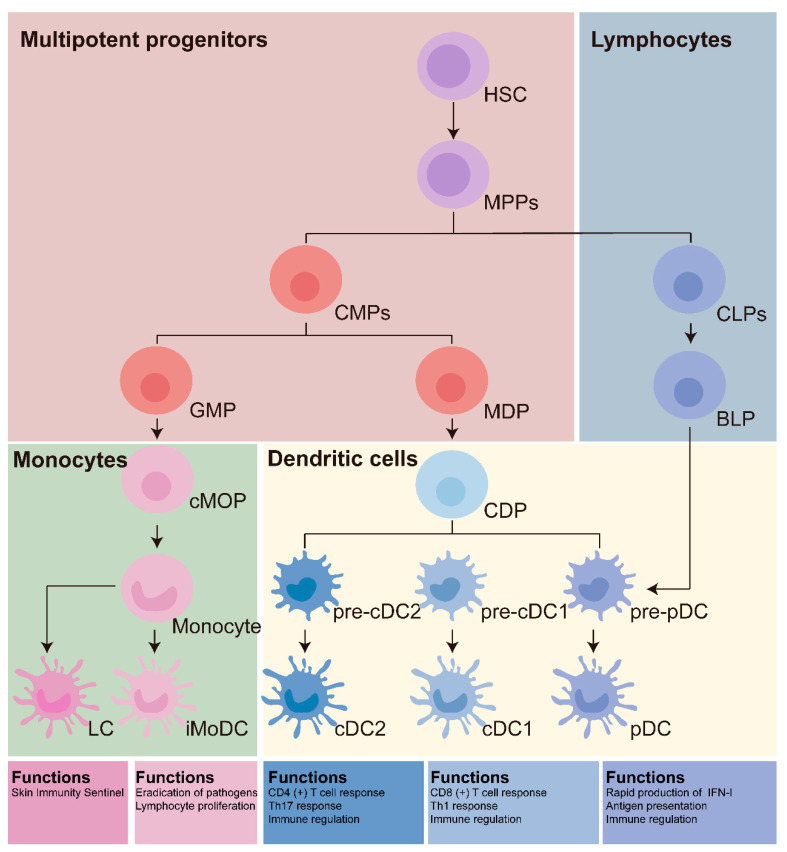
Model and function of DCs lineages development. The various types of dendritic cells (DCs) in normal physiological conditions, along with their origins and functions, are delineated. Hematopoietic stem cell (HSC)-derived multipotent progenitor cells (MPPs) can differentiate into common lymphoid progenitors (CLPs) and common myeloid progenitors (CMPs). Plasmacytoid dendritic cells (pDCs) originate from CLPs. CMPs further differentiate into granulocyte–monocyte progenitors (GMPs) and monocyte–dendritic cell progenitors (MDPs), with conventional dendritic cells cDC1 and cDC2 arising from their respective precursors, precursor cDC1 and precursor cDC2s. Dendritic cells derived from blood monocytes (iMoDCs) are generated through common monocyte progenitors (cMoPs). Langerhans cells (LCs), which are self-renewing phagocytes located in the epidermis, are traditionally classified within the DC family.

The postnatal DCs originate from the hematopoietic stem cells (HSCs) within the bone marrow [21,22]. The HSCs differentiate into common DC progenitors (CDPs), which subsequently mature into fully developed DCs [23,24]. Different cytokines and transcription factors present in the bone microenvironment influence the differentiation of DCs. Furthermore, monocytes from the bone marrow move toward peripheral tissues in reaction to inflammatory signals, where they have the potential to develop into DCs [25]. The family of transcription factors known as interferon regulatory factors (IRFs) is essential for the development and function of DCs. IRF3 and IRF4 significantly influence the maturation, migration, and functional regulation of DCs [26,27]. Recent studies have shown that IRF5 controls plasminogeny and antibody production through different antigenic triggers [28]. The absence of IRF8 hinders the maturation and activation of moDC and cDC1, in addition to affecting the proliferation of cDC1, as well as their ability to uptake antigens and transport them to lymphoid organs for the initiation of T cells [29]. Furthermore, the Notch and Wnt signaling pathways drive the functional maturation of DCs, which is accompanied by morphological changes such as the formation of dendritic protrusions [30,31,32]. The processes of monocyte and DC development and their functions in mouse are shown in Figure 1.

### 2.3. The Distribution and Function of DCs In Vivo

DCs are primarily localized in peripheral tissues, lymphoid organs, and other critical immune structures [33]. The DCs function as “sentinels” in peripheral tissues that are susceptible to infections, such as the skin, respiratory tract, gastrointestinal tract, and urogenital tract [34,35,36,37]. Using their dendritic processes, the DCs rapidly recognize and capture any foreign antigens in these tissues and undergo a maturation process that enhances their antigen-presenting capabilities. Following this, the fully developed DCs move to secondary lymphoid organs like the lymph nodes and spleen, where they trigger humoral and cell-mediated immune responses through interactions with CD4+ T cells and CD8+ T cells, respectively [38]. In the intestinal environment, DCs not only identify and eradicate pathogens but also establish immune tolerance by interacting with the intestinal epithelial cells [39]. This tolerance mechanism is crucial for maintaining intestinal homeostasis and preventing the immune system from attacking the resident microflora, which, in turn, averts the onset of inflammatory bowel disease, allergic reactions, and other immune-related disorders [40,41].

Certain DC subsets are more suited for vaccine development due to their unique properties. These DC types possess enhanced antigen-presenting capabilities, enabling them to effectively stimulate T-cell responses. They also exhibit a higher migratory potential, allowing them to efficiently transport antigens to lymph nodes where immune responses are initiated. Furthermore, specific DC subsets can be targeted to induce either Th1 or Th2 responses, depending on the type of vaccine required. This versatility allows for the tailored design of vaccines against various pathogens. Additionally, these DC types are relatively resistant to tolerization, making them less likely to induce immune tolerance and more likely to elicit protective immunity. Overall, these characteristics make certain DC subsets ideal candidates for vaccine development.

## 3. DCs and the Tumor Immune Micro-Environment

DCs play a significant role in anti-tumor immune surveillance and response. However, intra-tumoral DCs often exist in an immature or functionally impaired state due to the presence of TGF-β, IL-10, vascular endothelial growth factor (VEGF), and other immunosuppressive factors within the TME [42,43]. In addition, the unique physical and biochemical features of the TME, such as excessive collagen deposition in the extracellular matrix (ECM), hypoxia, and acidic metabolic byproducts, also aid in immune evasion by impairing the maturation, migration, and antigen-presenting functions of DCs [44].

High levels of IL-10 and TGF-β in the TME inhibit the functional maturation of DCs, leading to reduced expression of MHC-II and CD80/CD86, which directly diminishes their capacity to activate effector T cells [45,46]. Furthermore, melatonin substantially inhibits the capacity of DCs to produce IL-1β and IL-6 by obstructing the NF-κB signaling pathway, which, in turn, hinders the activation of Th1-type and Th17-type immune responses [47]. Likewise, IL-23 can diminish the antigen-presenting capability of DCs and the secretion of pro-inflammatory cytokines by inhibiting the STAT4 signaling pathway [48]. Additionally, VEGF has the ability to hinder the differentiation and maturation of DCs, while simultaneously facilitating angiogenesis in tumors [49,50,51]. The immunosuppressive cells in the TME, such as Tregs and MDSCs, also exert significant negative regulatory effects on the function of DCs [52]. Tregs stimulate the DCs to produce immunosuppressive molecules (such as IDO-1), either through the secretion of inhibitory cytokines or via direct contact [53]. Moreover, MDSCs have the ability to hinder the functional development of DCs through the secretion of reactive nitrogen species (RNS) and reactive oxygen species (ROS) [52]. Collectively, these factors create an immunosuppressive environment that is conducive to tumor growth.

Extensive cross-linking of collagen fibers in the tumor ECM causes tissue remodeling, resulting in a hardened and dense matrix that impedes the infiltration of DCs, eventually diminishing their ability to capture, process, and present antigens [44,54]. Moreover, collagen fibers modulate the phenotype and function by interacting with the surface integrin receptors, such as α2β1 integrin [55]. High levels of collagen within the TME may also induce an immunosuppressive phenotype in the DCs, thus inhibiting their ability to activate T cells and consequently weakening the anti-tumor immune response [54]. The antigen-presenting ability of DCs is notably compromised by the hypoxic condition within the tumor microenvironment (TME), mediated by the hypoxia-inducible factor 1-alpha (HIF-1α) [56]. Studies show that hypoxia inhibits the migration and maturation of DCs and prevents their effective entry into lymph nodes to initiate immune response [57]. Furthermore, the hypoxic environment can reprogram the metabolic pathways in the DCs, such as inhibiting oxidative phosphorylation, thereby compromising their energy supply and reducing their functional activity [58]. The large amounts of lactate produced during glycolysis directly suppress the function of DCs [59]. Lactate reduces intracellular pH levels, which hinders the release of pro-inflammatory cytokines and encourages the production of IL-33, thereby resulting in an immunosuppressive phenotype [60,61]. Additionally, lactate may hinder the movement of DCs from the tumor location to the lymph nodes [62].

In summary, DCs play a pivotal role in overcoming the immunosuppressive tumor microenvironment. First, by blocking the interaction between immunosuppressive cells and DCs, the inhibition of DC functions by immunosuppressive cells can be effectively alleviated. Second, improving the immunosuppressive factors within the tumor microenvironment involves regulating the cytokine milieu to reshape the immune balance, ameliorating the hypoxic condition, and suppressing the impact of metabolites, all of which contribute to enhancing the immune activity of DCs. In addition, enhancing the antigen-presenting capacity of DCs is achievable through optimizing their maturation and activation processes. This optimization can improve the efficiency of tumor antigen uptake, processing, and presentation. Moreover, the combination with immune checkpoint inhibitors can relieve the suppression of T-cells by immune checkpoints, synergistically boosting the ability of DCs to activate T-cells and thereby strengthening the anti-tumor immune response. Finally, nanotechnology can be employed to deliver antigens and adjuvants and target the tumor microenvironment. This approach enables precise delivery to the tumor site and DCs, enhances the uptake of tumor antigens by DCs, and stimulates a more robust immune response. By comprehensively implementing these strategies, the functions of DCs can be effectively enhanced, breaking through the barriers of the immunosuppressive tumor microenvironment and significantly increasing the intensity and persistence of the anti-tumor immune response. At present, research on DCs in overcoming the immunosuppressive tumor microenvironment is continuously advancing, opening up a new avenue for tumor treatment and offering hope for tumor cure.

## 4. Therapeutic Potential of DCs in Various Cancers

DCs have demonstrated remarkable therapeutic potential in various types of cancers. For instance, in lung cancer, DCs have been engineered to present tumor-associated antigens, thereby stimulating robust anti-tumor immune responses. Similarly, in breast cancer, DC-based vaccines have shown promise in eliciting specific T-cell responses against tumor cells. Furthermore, in melanoma, the adoptive transfer of tumor-infiltrating lymphocytes, which are often primed by DCs, has led to significant clinical responses. In colorectal cancer, DCs pulsed with tumor lysates have been used to induce systemic anti-tumor immunity. Moreover, in hepatocellular carcinoma and pancreatic cancer, DC-based immunotherapy strategies are being actively investigated to enhance patient outcomes. The versatility and plasticity of DCs make them powerful tools in the fight against cancer.

### 4.1. Lung Cancer

Lung cancer has the highest mortality rate globally among all malignancies [63]. Research has shown a significant decrease in the counts of dendritic cells both in the periphery and those infiltrating tumors in patients with lung cancer [64]. Additionally, DCs generally display an undeveloped phenotype marked by reduced expressions of MHC-II, CD80, and CD86. These immature DCs exhibit limited antigen uptake, which decreases their ability to activate T cells, thereby weakening the anti-tumor immune response. Furthermore, the excessive amounts of lactate secreted by lung cancer cells alter the metabolic state of DCs, which shifts the cells to a tolerogenic phenotype [60].

In recent times, a variety of strategies have been suggested by researchers to tackle the functional suppression of DCs in lung cancer. New investigations have examined the vital function of DCs in the context of lung cancer immunotherapy. By using multifunctional catalytic peroxide antigen-capturing nanosponges, researchers have been able to reprogram dysfunctional DCs, thereby enhancing remote immune therapy for lung metastasis [65]. Furthermore, engineered DCs that express CXCL9/10 not only facilitated T-cell activation but also considerably improved the effectiveness of immune checkpoint blockade in lung cancer [66]. In the treatment of advanced recurrent non-small cell lung cancer, PD-1 blockade-activated novel neoantigen-specific cellular therapy demonstrated substantial potential in cancer immunotherapy, exhibiting good safety and immunogenicity [67]. Additionally, the immune marker CD1C, associated with dendritic cells, has been demonstrated to act as a promising biomarker for forecasting prognosis and response to immunotherapy in patients with lung adenocarcinoma [68].

### 4.2. Breast Cancer

Breast cancer is one of the most prevalent malignancies among women [69]. In breast cancer tissues, the number of DCs is significantly reduced, constituting only about 20% of that in normal breast tissue [70]. These cells are predominantly in an immature or immune-tolerant state. Mature DCs are mainly located in the peritumoral regions [71]. Furthermore, in the tumor-draining lymph nodes of breast cancer patients, the aggregation of DCs is also decreased, with immature DCs still accounting for the majority [72]. Furthermore, breast cancer cells inhibit the differentiation and maturation of DCs by secreting IL-6, IL-10, IL-12, and prostaglandin E2 (PGE2), which downregulate co-stimulatory molecules [73,74]. In breast tumors, high levels of PD-L1 are frequently expressed by DCs, leading to the suppression of effector T cells and promoting immune evasion [75]. Research has also shown that there is a decrease in the quantity of peripheral DCs in patients with breast cancer, accompanied by a reduction in the expression levels of surface chemokine receptors like CCR7. This decline restricts the migration and antigen presentation capabilities of the DCs [76]. Research indicates that a lack of CCL19+ impairs the activation of CCR7+ CD8+ T cells, which subsequently diminishes the effectiveness of anti-PD-1 immunotherapy. Consequently, DCs expressing CCL19+ can improve the impact of anti-PD-L1 immunotherapy in the context of triple-negative breast cancer [77]. Furthermore, the activation of the CCR7/CCL21 axis by TGF-β1-induced EMT enhances the migration of breast cancer cells via the lymphatic system [78].

Immunotherapeutic strategies targeting DCs in breast cancer have shown encouraging results. Autologous DC vaccines can induce a Th-1 immune response, leading to significant anti-tumor effects against autologous breast cancer cells [79]. Tumor-derived exosomes, utilizing non-invasive photoacoustic imaging technology, stimulate DC activation, enhancing their migration to lymph nodes in mice and upregulating co-stimulatory molecules such as CCR7 and TNF-α [76,80]. Additionally, DCs that have been transfected with mRNA encoding tumor antigens are capable of eliciting strong T cell responses in both in vitro and in vivo settings, thereby promoting effective protective immunity and facilitating the progress of immunotherapy [81].

### 4.3. Melanoma

Melanoma is a highly immunogenic tumor, albeit with an immunosuppressive microenvironment that can suppress DC function. The DCs in melanoma patients often exhibit an immature phenotype with low surface expression of MHC and co-stimulatory signals, which hampers their ability to effectively activate anti-tumor T cells [45,82]. Vaccines based on DCs that carry gp100 or MART-1 antigens have demonstrated the ability to stimulate T cell responses specific to tumors; however, their effectiveness as standalone treatments is restricted [83,84]. The combination of DC vaccines with CTLA-4 and PD-1 inhibitors showed synergistic effects against melanoma by activating anti-tumor T cells and alleviating immune suppression [85,86]. Furthermore, studies have shown that iPSC-derived exosome-pulsed dendritic cell vaccines significantly enhance anti-tumor immunity against melanoma [87]. Furthermore, restoring tumor immunogenicity through the reprogramming of DCs presents a novel approach to enhancing immune responses. By adjusting the essential gene network of cDC1, researchers have transformed cancer cells into specialized antigen-presenting cells, which were subsequently administered to subcutaneous melanoma tumors. This innovative strategy successfully slowed tumor progression and increased survival rates in mice [88]. Additionally, research indicates that inhibiting the Polycomb repressor complex 2 (PRC2) leads to an increase in MHC-II expression, likely owing to enhanced chromatin accessibility of CIITA, which is the principal regulator of MHC-II. This underscores the promise of focusing on the Enhancer of zeste homolog 2 (EZH2), the functional enzymatic component of the Polycomb Repressive Complex 2 as a potential therapeutic approach to boosting the efficacy of immunotherapy [89].

### 4.4. Colorectal Cancer

The microenvironment of colorectal tumors is highly immunosuppressive, which is particularly evident in the functional inhibition of DCs. In the tissues of colorectal cancer patients, studies have found that various inhibitory cytokines secreted by the tumor and its associated stromal cells (such as VEGF, IL-10, TGF-β, etc.) interfere with the normal maturation process of DCs, leading to impaired antigen-presenting function [90]. This is manifested by a decrease in the infiltration of mature DCs (CD83^+^). At the same time, the proportion of immature DCs (CD1a^+^) increases. However, due to their weaker antigen-presenting function and lower expression levels of co-stimulatory molecules and MHC molecules, these immature DCs are unable to effectively activate T cells [91]. Studies suggest that combining DCs immunotherapy with cytokine-induced killer cell therapy may become an effective approach to control tumor growth in post-surgical gastric cancer (GC) and colorectal cancer (CRC) patients [92]. Musashi-2 facilitates the maturation and migration of DCs by influencing the post-translational modifications of HMGB1, which, in turn, boosts the infiltration of CD4+ and CD8+ T lymphocytes along with inflammatory responses, ultimately enhancing immune infiltration in colorectal cancer [93]. Additionally, DC-derived ROS activate the SENP3-IFI204 interaction, which promotes the activation of STING signaling in mice, thereby enhancing DC activity [94]. Arf1 ablation activates super-signal complexes in DCs, thereby boosting anti-tumor immunity [95]. Nanostars are nanoparticles with a star-shaped geometry, characterized by multiple sharp tips that enhance their interaction with light. These unique structures are particularly effective in photothermal therapy because they can efficiently convert absorbed light into heat. Photothermal therapy utilizing gold nanostars, when paired with DC-based immunotherapy, successfully addresses the resistance often seen in colon cancer cells [96]. In addition, the use of chemotherapy agents, like 5-FU, alongside DCs has demonstrated an improvement in antigen-specific immune responses, though their effectiveness requires additional refinement [97]. These results underscore the promise of this integrated treatment strategy for colorectal cancer management.

### 4.5. Hepatocellular Carcinoma

The microenvironment of hepatocellular carcinoma (HCC) suppresses the functionality of DCs through various mechanisms. Research indicates that exosomes from DCs altered with rAAV/AFP can provoke a targeted T cell-mediated immune response against HCC [98]. In an experimental mouse model of HCC, inhibiting the cell surface protein CD47 activates CD103+ DCs, which facilitates the recruitment and stimulation of natural killer cells, thereby boosting anti-tumor immunity [99]. Furthermore, dendritic cell-derived exosomes (DEX) that are coated with peptides targeting HCC (P47-P), epitopes of alpha-fetoprotein (AFP212-A2), and domains of high-mobility group nucleosome-binding protein 1 (N1ND-N) function as immune adjuvants, eliciting specific immune responses against tumors in HCC via exosome-anchored peptides [100]. Furthermore, STING agonist-based hydrogels combined with incomplete radiofrequency ablation synergistically enhance DC antigen presentation, effectively activating immune responses for hepatocellular carcinoma treatment [101]. Radiofrequency ablation, or radiofrequency ablation, is a minimally invasive interventional technique. This technique is mainly used to treat diseases such as tumors and arrhythmias and has the advantages of small trauma, quick recovery, and an exact curative effect.

### 4.6. Pancreatic Cancer

Pancreatic cancer is characterized by high malignancy, low survival rates, poor prognosis, and a significant immunosuppressive environment. In the tumor microenvironment, increased concentrations of TGF-β, IL-10, and PGE2 hinder the effective maturation of DCs. Additionally, the dense collagen matrix restricts DC migration and suppresses its metabolism and activity through interactions with integrin receptors. To address these challenges, DC vaccines targeting KRAS-mutated pancreatic tumors have been developed [102,103], and DC-based immunotherapy has been shown to induce T cell responses against pancreatic cancer antigens, offering the potential to prevent disease recurrence [104]. Research also showed that Zymogen granule protein 16 (ZG16) enhances DC maturation by inducing CD40, contributing to anti-tumor immunity in pancreatic cancer [105]. Moreover, gemcitabine-treated pancreatic cancer cell-conditioned media stimulates DC maturation, alters tumor cell immunogenicity, and induces specific cytotoxic T lymphocyte anti-tumor activity [106]. The joint action of ginsenoside Rh2 and gemcitabine improves DC immune responses to pancreatic cancer via the CARD9-BCL10-MALT1 / NF-κB signaling pathway [107]. Additionally, overcoming immune resistance in pancreatic cancer has been achieved by co-delivering CCR2 antagonists using STING-activated gemcitabine nanocarriers [108].

However, the immune evasion mechanisms in pancreatic cancer remain complex. The absence of caspase recruitment domain-containing protein 9 in cells of the innate immune system hinders the maturation of DCs by means of SLC6A8-mediated transport of creatine, which consequently facilitates the progression of pancreatic cancer [109]. These studies highlight the crucial role of DC function and maturation in pancreatic cancer immunotherapy, suggesting that interventions targeting these mechanisms may provide effective therapeutic strategies in the future.

There are many reasons why DC vaccines are limited in cancer treatment. First, DC vaccines rely on the effective activation and maturation of dendritic cells in patients, but cancer patients often have immunosuppression that affects DC function. Second, immunosuppressive factors in the tumor microenvironment, such as TGF-β and IL-10, inhibit the immune response induced by the DC vaccine. Furthermore, tumor cells may circumvent the attack of the DC vaccine by down-regulating antigen expression or generating immune escape mechanisms. In addition, the preparation and distribution of DC vaccines are complex, costly, and require individual customization, which limits their widespread use.

## 5. Clinical Applications of DC Vaccines

### 5.1. Mechanism of Action of DC Vaccines

**Figure 2:**
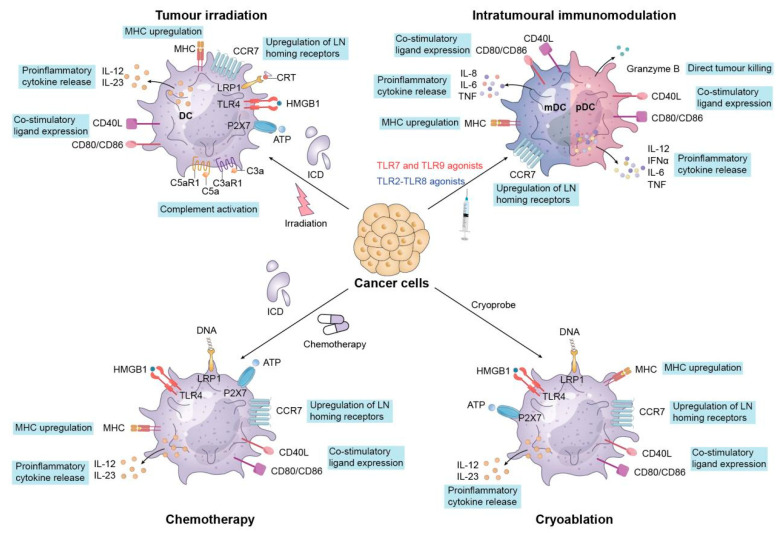
Mechanisms underlying tumor immunogenicity triggered by conventional treatment approaches. 1. Tumor Irradiation: Exposure to irradiation leads to immunogenic cell death (ICD) in tumor cells, resulting in the release of danger-associated molecular patterns (DAMPs), including ATP, HMGB1, and calreticulin (CRT). These substances stimulate DCs through receptors such as TLR4 and P2X7, enhancing the processes of antigen acquisition and the upregulation of MHC. 2. Intratumoral Immunomodulation: Direct administration of TLR2 and TLR9 agonists within the tumor microenvironment triggers DC and plasmacytoid dendritic cell (pDC) activation. These agonists stimulate the release of proinflammatory cytokines such as IL-12, IL-6, and TNF, as well as cytotoxic molecules like granzyme B. 3. Chemotherapy: Certain chemotherapeutic agents can induce ICD, characterized by the release of DAMPs (HMGB1, ATP) and exposure of CRT. This signals DC activation and enhances antigen presentation through MHC molecules. 4. Cryoablation (Cryotherapy): Cryoablation causes tumor cell lysis, releasing intracellular contents such as DNA, ATP, and HMGB1. These molecules interact with receptors like TLR4 and P2X7 on DCs, triggering immune activation pathways similar to those seen in other ICD-inducing therapies.

The transfer of DCs that are loaded with tumor antigens can trigger specific immune responses against tumors in the host. Initially, DCs are obtained from the peripheral blood mononuclear cells (PBMCs) of patients and are then cultured with GM-CSF and IL-4 to promote their maturation [110]. Subsequently, the fully developed DCs encounter tumor antigens, which may include entire protein extracts derived from tumor cells, specific tumor antigens like CEA and HER2, or altered neoantigens [111,112]. The DCs then ingest and process these antigens through phagocytic receptors or Fc receptors and present the antigenic peptides via MHC molecules [113]. Finally, cytokines or immune stimulants such as TLR agonists are used to activate the DCs, enhance their antigen presentation ability, and promote their migration to lymph nodes, thereby effectively inducing specific immune responses [114]. After being infused into the host, DCs present tumor antigens to CD8+ cytotoxic T cells and CD4+ helper T cells via MHC molecules, thereby triggering specific immune responses against tumors. Additionally, the activation of DCs can enhance Th1 immune responses and lead to the secretion of cytokines like IFN-γ, which further boosts the activity of cytotoxic T cells [115]. Finally, the infused DC tumor vaccine regulates the activation state of DCs, enhances their antigen presentation ability, overcomes the immune escape mechanism in the TME, and strengthens anti-tumor immune responses (Figure 2).

### 5.2. DC Vaccines in Clinical Testing Phase

Trepiakas et al. conducted a clinical trial to evaluate the use of DC vaccines in melanoma patients [116]. Autologous mature DCs derived from monocytes were treated with peptides derived from p53, survivin, and telomerase (for HLA-A2+ patients) or with tumor lysates from autologous/allogeneic sources (for HLA-A2− patients), along with low-dose IL-2 and IFN-α2b. In their research, 24% of patients suffering from metastatic disease showed stable disease (SD) related to the treatment. Further evaluation of these individuals indicated improved overall survival (OS) and progression-free survival (PFS). Moreover, the level of regulatory T cells (Tregs) was notably lower in patients exhibiting SD when contrasted with those with progressive disease (PD). This observation implies that the DC vaccine may have influenced immunosuppressive cells, leading to its anti-tumor effects. Similarly, Cho et al. assessed the effectiveness of the autologous DC vaccine in patients with glioblastoma multiforme (GBM) [117]. The 1-, 2-, and 3-year survival rates for patients receiving the vaccine were 88.9%, 44.4%, and 16.7%, respectively, while those in the placebo group had rates of 75%, 18.8%, and 0%. The median overall survival for the vaccine cohort was reported at 31.9 months, compared to 15 months for the control cohort (*p* < 0.002). Additionally, the median progression-free survival for the vaccine and control groups was 8.5 months and 8 months, respectively (*p* = 0.075). Consequently, the use of autologous DC vaccines for glioblastoma multiforme may lead to a significant enhancement in clinical outcomes for glioma patients.

Inogés et al. [118] demonstrated the feasibility and safety of tumor lysate-pulsed autologous DC vaccine in glioma patients who underwent tumor resection and combined radiotherapy and chemotherapy. A total of 32 patients of [median age 61 years (42–70 years)] were included in the study, of whom 1 failed screening due to inadequate resection. No significant adverse effects associated with immunotherapy were observed. A rise in immune response specific to tumors, evidenced by T cell proliferation and cytokine production, was noted in 11 patients following their vaccination. These findings suggest that DC vaccines may be beneficial during various phases of tumor development.

Baek et al. [119] treated six renal cancer patients and four patients with breast cancer who received DC vaccine treatment. Two infusions of autologous CD34+, HSC-derived, which had been differentiated with GM-CSF and IFN-γ and pulsed with autologous tumor lysate and KLH, were administered four weeks apart. After each infusion, low-dose (200 MIU) IL-2 was introduced as an immune adjuvant for 14 consecutive days. This combination therapy was found to be well-tolerated and effectively stimulated antigen-specific immunity while reducing immunosuppressive cues, which resulted in clinical responses. In a phase I/II trial, Baek et al. determined the therapeutic efficacy of DC vaccines combined with IL-2 against ovarian cancer [120]. Ten patients with minimal residual disease who had undergone primary treatment were enrolled. A complete remission (CR) was achieved by three patients after they were administered the DC vaccine, with no recurrence for durations of 83, 80.9, and 38.2 months, respectively. In addition, one patient with SD experienced a complete regression of the tumor after receiving the DC vaccine, maintaining this state for 50.8 months before recurrence occurred. In contrast, two patients who exhibited partial remission (PR) did not benefit from the DC vaccine and went on to experience disease progression. Remarkably, significant changes in immunological markers were noted in the three long-term survivors without disease, including enhanced activity of natural killer (NK) cells, an increase in interferon-gamma (IFN-γ)-secreting T cells, elevated levels of immunostimulatory cytokines, and decreased concentrations of immunosuppressive factors. These observations provide strong evidence that DC vaccination has the potential to enhance the prognosis of patients with ovarian cancer by fostering vigorous anti-tumor immune responses.

A phase II clinical trial on DC-based immunotherapy for refractory solid tumors was carried out by Bapsy et al. [120]. In total, 51 patients suffering from refractory cancer, each with a life expectancy of less than three months, were enrolled from six different centers. Autologous monocytes were differentiated into DCs in vitro, after which the mature DCs were reinfused into the participants. The therapeutic efficacy was assessed in 38 patients during the follow-up period. The objective response rate (ORR), as determined by the Response Evaluation Criteria in Solid Tumors (RECIST), was found to be 28.9% (11 out of 38) (90%CI 17.2–;43.3), whereas the immune-related response rate was reported at 42.1% (16 out of 38) (90%CI 28.5–56.7). The median duration to treatment progression was over 9 weeks, and the median OS lasted 397 days. Additionally, levels of IFN-γ increased following DC vaccination, although this change was not statistically significant. These results suggest that DC-based immunotherapy represents a safe therapeutic option for patients with refractory solid tumors [121].

While autologous DC transplantation has achieved significant clinical efficacy against various cancers, DC vaccines prepared using electroporation dendritic cells (EP-DCs) have been similarly effective. Kamigaki et al. developed an EP-DC vaccine by incorporating autologous tumor lysate into DCs using a closed-current electroporation system. This vaccine was evaluated in 41 patients suffering from various solid tumors, yielding an ORR of 4.9% (2/41) for CR and PR combined, while the clinical benefit rate—which includes CR, PR, and sustained SD—was noted to be 31.7% (13/41). Moreover, a positive result in the delayed-type hypersensitivity (DTH) test was observed in the majority of patients who experienced long-term SD, with the DTH positivity rate among those who derived clinical benefit reaching 91.7% (11/12) [122].

DC vaccines have also shown potential in the treatment of hematological tumors. Di Nicol et al. [123] treated 18 patients with relapsed indolent non-Hodgkin lymphoma (NHL)with autologous tumor cell-loaded killer DC vaccine. A total of six patients showed measurable clinical responses; among them, three achieved sustained CR while the other three had PR over a median follow-up period of 50.5 months. Additionally, eight patients maintained SD, whereas four patients showed PD. Moreover, DC vaccination markedly improved the anti-tumor activity of IFN-γ-producing T cells in those with partial responses. Overall, these results indicate that DC vaccines loaded with tumor antigens can stimulate both T cell and B cell immune responses, leading to clinical benefits for patients with indolent NHL.

Caballero-Baños et al. conducted a clinical trial to evaluate the efficacy of an autologous tumor lysate DC (ADC) vaccine in the treatment of metastatic CRC. Regrettably, the ADC vaccine failed to demonstrate a meaningful effect on OS or PFS among the patients [124], highlighting the shortcomings of DC vaccines. It is essential to investigate various strategies to boost the effectiveness of DC vaccines, including the enhancement of DC function and anti-tumor immune responses, as well as the formulation of combination therapies.

### 5.3. DC Vaccine Combined with Chemotherapy and Radiotherapy in the Treatment of Tumor

Due to the restricted clinical effectiveness of DC vaccines, various research teams have investigated the results of integrating DCs with additional anti-cancer treatments. Preclinical evidence suggests that chemotherapy may have immunogenic effects and augment the effects of immunotherapy. In a preliminary investigation, Lesterhuis and colleagues [125] assessed how standard adjuvant oxaliplatin/capecitabine chemotherapy influences both specific and non-specific immune responses generated by a pulse-processed DC vaccine that was loaded with keyhole limpet hemocyanin (KLH) and CEA peptides in patients diagnosed with stage III CRC. Out of the seven subjects, four exhibited functional T-cell responses specific to CEA as assessed by the DTH skin test. Furthermore, an increase in non-specific T-cell responses was noted following oxaliplatin administration. Although chemotherapy did not affect the KLH-specific T-cell responses, a reduction in B-cell responses was recorded. The findings of this study indicate that combining chemotherapy with a DC vaccine may enhance anti-tumor immune responses in patients with CRC [124].

There is insufficient clinical evidence to support the efficacy of DC vaccines combined with radiotherapy in the treatment of solid tumors. According to Huang et al., cancer vaccines based on induced pluripotent stem cells (iPSCs) that are modified with neoantigens can trigger T cell responses specific to these neoantigens, helping to eliminate cancer cells and markedly enhance the effectiveness of radiotherapy for poorly immunogenic CRC and triple-negative breast cancer (TNBC) [126]. The neoantigen-enhanced iPSCs (NA-iPSCs) were prepared by cloning the neoantigen genes in AAV2 vectors. The combination of NA-iPSC vaccines and irradiation was tested in a mouse model of microsatellite-stable CRC, and 60% of the mice achieved CR post-treatment. Moreover, spleen cells from the treated mice generated elevated amounts of IFN-γ upon exposure to neoantigens and demonstrated enhanced cytotoxicity against cancer cells, indicating that the combination therapy may elicit a more robust neoantigen-specific T cell response. In summary, the combined use of DC vaccines with chemotherapy or radiotherapy may provide a new treatment strategy for tumors with poor immunogenicity, such as CRC and TNBC, and thus warrant further research and clinical verification.

## 6. Recent Developments in Nanotechnology for DCs Applications

Biomimetic nanoparticles (NPs) are designed to mimic natural biological structures and functions, offering unique advantages in the field of dendritic cell (DC)--based immunotherapy. These NPs can be engineered to specifically target DCs, enhance their antigen-presenting capabilities, and stimulate robust immune responses. One of the key advantages of biomimetic NPs lies in their ability to improve the biodistribution and cellular uptake by DCs. By mimicking natural ligands or receptors, these NPs can effectively bind to specific receptors on DC surfaces, facilitating efficient internalization and processing of antigens. Furthermore, biomimetic NPs can protect antigens from degradation, ensuring their stability and bioavailability within the DC compartment. This, in turn, enhances the presentation of tumor-associated antigens to T cells, leading to a more potent and specific immune response against cancer cells. Additionally, these NPs can be functionalized with adjuvants or immunostimulatory molecules to further potentiate the immune response, making them a versatile platform for DC-based vaccine development.

### 6.1. Designing Biomimetic NPs for DC Vaccines

**Figure 3:**
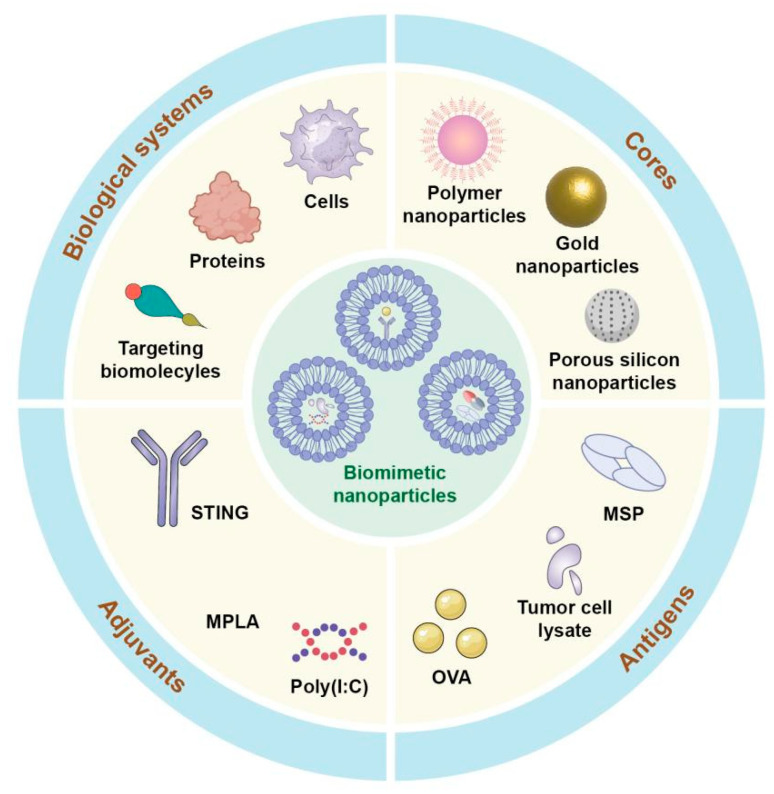
Schematic representation of the dendritic cell vaccination approach utilizing biomimetic nanoparticles. 1. Biological Systems: Biomimetic nanoparticles can incorporate biological components such as proteins, whole cells, or targeting biomolecules to mimic natural biological systems. These components facilitate targeted delivery and improve interaction with dendritic cells by leveraging biological recognition pathways. 2. Cores: The nanoparticles’ cores are composed of materials that ensure structural integrity and functionality. Examples include polymer nanoparticles, gold nanoparticles, porous silicon nanoparticles, and mesoporous silica particles (MSP). These core materials are selected based on their biocompatibility, antigen-loading capacity, and ability to enhance antigen stability and presentation. 3. Antigens: Tumor-specific antigens or model antigens (e.g., tumor cell lysates or ovalbumin [OVA]) are incorporated into the nanoparticles to activate dendritic cells. These antigens are critical for eliciting an adaptive immune response as they are processed and presented by DCs to stimulate T cell-mediated immunity. 4. Adjuvants: Immunostimulatory molecules such as STING agonists, MPLA (monophosphoryl lipid A), or Poly (I:C) are included to enhance the activation and maturation of dendritic cells. These adjuvants act as danger signals, mimicking microbial infections and boosting the immune system’s response to the delivered antigens.

The design of biomimetic NPs for DC vaccines focuses on enhancing targeting specificity by integrating specific molecules, simulating the behavior of APCs to directly activate T cells, increasing biocompatibility by using natural materials, combining antigen delivery and adjuvant properties to optimize immune activation, and prolonging their in vivo circulation to ensure continuous immune stimulation [127]. The DCs can recognize and bind to specific ligands or antibody-modified NPs through surface receptors, such as C-type lectin receptors, Fc receptors, etc. Through receptor-mediated endocytosis, DCs ingest these NPs and activate immune responses. Thus, the incorporation of suitable targeting ligands, such as carbohydrates, peptides, or antibodies, into the NPs can increase their uptake by the DCs and improve vaccine function [128]. Furthermore, NPs can be designed to mimic the antigen presentation function of DCs and directly interact with T cells by modifying their surface with antigen-peptide complexes and co-stimulatory molecules [129]. NPs are typically fabricated from biocompatible materials such as lipids, polymers, or other natural substances. These materials are characterized by low immunogenicity, favorable biodegradability, and high compatibility with biological systems. Consequently, NPs constructed from these substances can significantly mitigate the risk of immune rejection. Moreover, they enhance the stability of nanoparticles within the body and prolong their circulation time, thereby improving therapeutic efficacy and bioavailability [130,131]. Although the integration of these complex functions in nanoscale systems currently faces challenges in the manufacturing process, innovative biomimetic NPs offer significant potential to augment the efficacy of cancer immunotherapy [132,133,134] (Figure 3). Part A shows nano-carriers improved targeting efficiency to DCs, with a significant increase in uptake compared to controls. Part B focuses on nano-carriers enhanced stability and biocompatibility, with preclinical studies indicating minimal toxicity and sustained release. Part C demonstrates nano-carriers ability to modulate DC maturation and activation, leading to stronger immune responses. Preclinical data show increased cytokine production and T-cell proliferation after DC stimulation with nano-carrier-loaded antigens. Lastly, Part D illustrates the potential of nano-carriers to enhance therapeutic payload delivery to specific DC subsets for precise immune modulation.

### 6.2. Molecular Mechanism of Biomimetic NPs Enhancing the Action of DC Vaccine

Biomimetic NPs enhance the effectiveness of the DC vaccine through immune regulation, targeted delivery, and improved antigen presentation. Biomimetic NPs are designed to mimic the structure and function of pathogens or endogenous immune signals. It usually leads to the upregulation of co-stimulatory molecules (such as CD80, CD86, CD40) and an increase in cytokine secretion (such as IL-12, IL-6, TNF-α), thus promoting the maturation of DC and enhancing the immune response [135]. Biomimetic NPs are designed to optimize the loading, stability, and release of antigens. When DCs internalize these NPs, they can deliver antigens directly to the endocytic compartments of DCs, promoting efficient processing and presentation via MHC (major histocompatibility complex) molecules. Moreover, it can improve the persistence and release profile of the antigens, ensuring that DCs are exposed to a sustained supply of antigens and are more likely to prime T cells effectively [136]. The surface properties of biosimilar nanoparticles can be activated by adding ligands or antibodies to specifically target dendritic cell receptors, activating both innate and adaptive immune responses [137]. DCs play a crucial role in driving Th1 (helper T cell) and CTL (cytotoxic T lymphocyte) responses. By enhancing antigen presentation and cytokine release, biomimetic NPs can direct DCs to prime these T cell subsets, which are particularly important in the context of vaccines against cancer or infections [138].

### 6.3. Advantages and Limitations of Combining Nanotechnology with DCs

A recent study [139] reported the synthesis of “Mini DCs”, wherein the membranes extracted from the DCs of ovarian cancer patients were coated with IL-2-loaded PLGA NPs. The Mini DCs expressed the membrane proteins with DCs, such as MHC, CD40, and CD86, which endowed them with the ability to activate T cells and overcome immunosuppressive factors in the TME. Meng et al. [140] synthesized stable microspheres MS(O10H6) loaded with a plasmid encoding mouse IL-10 (pIL-10), and transfected DCs with the MS(O10H6)-pIL-10 complexes to regulate their alloreactivity. These genetically modified DCs significantly reduced the proliferation of allogeneic CD4+ and CD8+ T cells induced in vitro. In an in vivo experiment using cells embedded in Matrigel as a transplant model, DCs transfected with MS(O10H6)-pIL-10 complexes were found to inhibit the infiltration of host cells. These results indicate that self-assembled MS(O10H6) is an efficient plasmid delivery tool for regulating DC-dependent allogeneic T cell responses.

While bionic nanotechnology provides excellent biocompatibility, minimal toxicity, robust spatiotemporal control, and immune activation on demand, there are still unavoidable challenges in its clinical translation. Initially, the comprehensive safety of bionic nanoparticles requires assessment through clinical trials [141]. Furthermore, the complexity of biomimetic approaches frequently arises from the integration of various functions, presenting a significant challenge to affordable manufacturing and large-scale production. It will be imperative to balance this functional complexity with a simple vaccine design structure for successful clinical translation [142]. Finally, adapting the current production scale of nano-DC vaccines to a high-throughput workflow will require a significant investment of time and resources [143,144]. Nevertheless, biomimetic nanotechnology has immense potential to open up a path for the development of a new generation of DC vaccines.

## 7. Summary and Future Prospects

DCs are highly promising in the realm of tumor immunotherapy because of their capability to present antigens to immune effector cells. Despite numerous studies indicating that DCs can provoke anti-tumor immune responses, there are considerable obstacles to the clinical application of DC-based tumor biotherapy. This is largely attributed to immunosuppressive factors present within the TME, which significantly impair the anti-tumor activity of DCs and restrict their therapeutic effectiveness. Moreover, DCs’ biological origins—such as peripheral blood and bone marrow—and the protocols for in vitro differentiation and activation play a crucial role in determining their efficacy against tumors in clinical scenarios. Additionally, the capability of DCs to present specific tumor antigens, elude immune tolerance, and identify tumor cells is also a critical factor in assessing their therapeutic potential. Lastly, the effectiveness of DC vaccines can vary greatly among individual patients, with the overall outcomes remaining somewhat constrained. Therefore, the future research directions for DC-based vaccines would be to optimize DC function, neutralize the immunosuppressive TME, and improve treatment efficiency by incorporating other immunotherapies. The technology of gene editing may be employed to improve the capacity of DCs to showcase tumor antigens, thereby boosting the immune responses initiated by DCs. Furthermore, the use of immune checkpoint inhibitors has the potential to enhance the clinical effectiveness of DC vaccines by counteracting the immunosuppressive signals present in the TME [145]. Furthermore, personalization and diversification of DC vaccines will also be a major research focus, as DC vaccines developed as per the tumor characteristics and immune systems of individual patients may improve therapeutic effects. Likewise, using tumor-derived antigens in combination with tumor-associated antigens may also improve therapeutic efficacy [146]. Finally, auxiliary immune factors such as interleukins, IFNs, etc., may enhance the immunogenicity of DC vaccines, resulting in more intense and persistent anti-tumor responses.

## 8. Conclusions

This article reviews the application of DC vaccines in cancer treatment and their promising prospects. The paper elaborates on the mechanism of action of DC vaccines and their clinical trial results in various types of cancer. By extracting DC cells from patients and training them, they can precisely recognize cancer cells and convey this information to T cells, thus achieving precise targeting of cancer cells. Multiple studies have shown that DC vaccines demonstrate safety and efficacy in treating various cancers, such as breast cancer, pancreatic cancer, lung cancer, and more, offering new treatment options for patients. Additionally, the article mentions some advantages of DC vaccines, such as precise and efficient tumor cell clearance, continuous mobilization of cytokines within the body to participate in tumor cell clearance, and long-term effects with memory capabilities. These advantages give DC vaccines an important role in cancer immunotherapy. With technological advancements and further research, DC vaccines are expected to become a significant means of cancer treatment in the future, bringing new hope to patients.
